# The relationship between cholesterol levels and thyroid eye disease

**DOI:** 10.1530/ETJ-24-0133

**Published:** 2025-02-03

**Authors:** Caroline Cardo, Roberto Bernardo Santos, Ana Beatriz Pinotti Pedro Miklos, Sabrina Barbosa Jaconis, João Hamilton Romaldini, Danilo Villagelin

**Affiliations:** ^1^ Postgraduate Course Internal Medicine, Campinas State University, Campinas. Brazil. Endocrinology and Metabolism, Hospital PUC-Campinas, Campinas, Brazil; ^2^ Endocrinology and Metabolism, Hospital PUC-Campinas, Campinas, Brazil; ^3^ Endocrinology and Metabolism, São Paulo State Public Servant Hospital (IAMSPE), São Paulo, Brazil; ^4^ Internal Medicine, Hospital PUC-Campinas, Campinas, Brazil; ^5^ Postgraduate Course Internal Medicine, Campinas State University, Campinas., Brazil. Medical School, Pontifical Catholic University of Campinas, Campinas, Brazil

**Keywords:** thyroid eye disease, cholesterol, Graves’ disease, hyperthyroidism

## Abstract

**Background:**

Thyroid eye disease (TED) is the most prevalent extrathyroidal manifestation of Graves’ disease (GD). Emerging evidence suggests a relationship between elevated total and low-density lipoprotein (LDL) cholesterol levels and TED. This study aimed to investigate this correlation in the Brazilian population by analyzing data from two tertiary care centers.

**Methods:**

Data were collected from GD patients treated with methimazole between 1999 and 2021, excluding those receiving other treatments. Laboratory results and information on smoking habits, statin use and medications affecting lipid profiles during the euthyroid state were analyzed.

**Results:**

Smoking and elevated LDL cholesterol levels were significantly associated with TED activity and severity. Logistic regression revealed correlations between higher LDL cholesterol, total cholesterol and increased clinical activity score (*P* < 0.01, OR: 1.012, 95% CI: 1.003–1.021; *P* < 0.01, OR: 1.010, 95% CI: 1.002–1.018). These were also associated with more severe disease forms as defined by EUGOGO (*P* < 0.01, OR: 1.015, 95% CI: 1.006–1.024; *P* < 0.005, OR: 1.011, 95% CI: 1.004–1.019). Multiple regression confirmed that TED activity was significantly correlated with LDL cholesterol (*P* < 0.01) and smoking status (*P* < 0.01). Disease severity was associated with reduced HDL cholesterol (*P* < 0.05, OR: 0.973, 95% CI: 0.948–0.999), elevated LDL cholesterol (*P* < 0.005, OR: 1.013, 95% CI: 1.004–1.023) and active smoking (*P* < 0.05, OR: 2.881, 95% CI: 1.190–6.971).

**Conclusion:**

Elevated LDL cholesterol may serve as a potential indicator of TED. Further research is needed to determine whether lipid-lowering interventions could reduce TED risk or improve its management.

## Introduction

Thyroid eye disease (TED) is an autoimmune disorder that affects the orbital tissues and is present in approximately 30–40% of individuals diagnosed with Graves’ disease (GD) (1). Various factors contribute to an elevated risk of TED, including thyroid dysfunction, smoking, radioiodine therapy, male gender, advanced age and specific genetic and demographic characteristics.

The pathogenesis of TED is complex and involves multiple interrelated mechanisms that are not yet fully understood (1). The development of TED begins with the activation of specific orbital fibroblasts, known as preadipocytes, mediated by thyroid receptor antibodies (TRAb). These preadipocytes subsequently differentiate into adipocytes, which increases the expression of thyrotropin receptors (1, 2, 3). Simultaneously, cytokines such as interferon-gamma and tumor necrosis factor (TNF) stimulate a distinct subset of orbital fibroblasts that express the Thy-1 antigen, resulting in elevated hyaluronan production (4).

Furthermore, the activation of the insulin-like growth factor-1 receptor (IGF1-R) on orbital fibroblasts plays a critical role by triggering the release of chemokines, notably interleukin-16 and RANTES. These chemokines facilitate the recruitment of activated T cells and other immune cells into the orbital region. T cells expressing CD154 interact directly with orbital fibroblasts, forming CD40–CD154 complexes. Oxidative stress also represents a key process in TED pathogenesis (4).

Activation of both receptors simultaneously leads to a synergistic increase in DNA synthesis and cell proliferation compared to the effects of each agonist alone (5). Thyroid-stimulating hormone (TSH) triggers monoubiquitination of insulin receptor substrate-2 in thyroid epithelial cells, thereby enhancing IGF-I signaling and its mitogenic activity (6, 7). Both IGF-I and TSH elevate nuclear β-catenin content, thus promoting Wnt-dependent proliferation of thyroid cells (8). IGF-I can independently induce proliferation in rat thyroid cells, suggesting it may also play a significant role as a regulator of thyroid growth compared to TSH (9).

The symptoms of TED can vary, but they are typically marked by inflammation, bulging eyes and double vision (10). Mild cases are more prevalent than severe ones, which may involve the optic nerve and impair vision (2, 10).

Choosing the proper treatment for TED is personalized based on the disease’s activity and severity. The clinical activity score (CAS) (11) and the EUGOGO (European Group on Graves’ Orbitopathy) classification (12) are tools used for this assessment.

The challenge lies in categorizing and comprehending the characteristics of the population affected by TED. This is crucial in exploring new treatment options for distinct patient groups, especially those with prevalent inflammation. In addition, it is essential to understand potential risk factors linked to these groups in different populations to assist future research. This study aims to investigate the association between cholesterol levels and TED within the Brazilian population, presenting population characteristics through analyses conducted at two tertiary care centers.

## Materials and methods

This study undertook a retrospective analysis of clinical and laboratory data from patients who received treatment for GD. Data were extracted from medical records at two tertiary centers spanning the years 1999–2021.

Patients included in the study were those who maintained a euthyroid state during clinical and laboratory evaluations and had been treated for GD with methimazole.

The presence of TED was assessed clinically using both the CAS (11) and the EUGOGO (10, 13) classification. The CAS assigns one point for each of the following symptoms: spontaneous retrobulbar pain, pain on upward or lateral gaze, redness of the eyelids or conjunctiva, eyelid swelling, caruncle inflammation and conjunctival edema (chemosis). A CAS of three or more points is indicative of active disease. In addition, the EUGOGO (10, 12) scale was utilized to evaluate disease severity.

In the EUGOGO classification, patients with mild orbitopathy exhibit features that minimally interfere with daily activities and do not necessitate immunosuppressive or surgical intervention. These patients typically present with one or more of the following symptoms: eyelid retraction of less than 2 mm, mild soft tissue involvement, exophthalmos less than 3 mm above the normal range (adjusted for race and sex), transient or absent diplopia and corneal exposure responsive to lubrication (10).

Moderate-to-severe TED includes patients who, while not at immediate risk of vision loss, experience a significant impact on the quality of life due to their ocular condition. The severity of these symptoms may warrant immunosuppressive therapy (if active) or surgical intervention (if inactive). Such patients typically present with one or more of the following signs: eyelid retraction of 2 mm or more, moderate-to-severe soft tissue involvement, exophthalmos of 3 mm or more above the racial and sex-specific norm and intermittent or constant diplopia.

Patients with sight-threatening TED are those at risk of reduced visual acuity, often associated with dysthyroid optic neuropathy (DON) and/or corneal breakdown (11, 12). For this study, these evaluations were conducted by authors RBS, ABPPM and DV.

During the euthyroid state at the time of laboratory evaluation, serum levels of thyrotropin (TSH), free thyroxine (fT4), TRAb, thyroglobulin antibodies (TgAb), thyroid peroxidase antibodies (TPOAb), total cholesterol (TC), cholesterol fractions and triglycerides were measured. TSH, fT4, TgAb and TPOAb serum concentrations were determined using chemiluminescent assays (DPC immulite system). The reference ranges were 0.27–4.2 mIU/L for TSH, 0.93–1.7 ng/dL for fT4, >40 IU/mL for TgAb and >35 IU/mL for TPOAb.

Over the years, different methods were employed for TRAb determination. From 1980 to 1991, an in-house assay was used, which measured the thyroid-stimulating antibody activity through cyclic AMP stimulation in human thyroid plasma membranes, with a positive TSAb reference of <128%. Between 1992 and 2008, a radioreceptor assay (RSR Ltd, UK) was first used, followed by a second-generation ELISA assay (RSR Ltd) with a normal range of <10 IU/L. More recently, a human monoclonal assay (M22 antibody) from Siemens Immulite-TSI was adopted, with normal values defined as <0.175 IU/L. To avoid confounding factors, antithyroid antibodies were reported as either positive or negative (14, 15).

Smoking status was also evaluated, and ultrasound was used to measure thyroid volume, with a reference range of 6–15 mL for a healthy thyroid gland.

Patients were excluded from the study if they were diagnosed with causes of hyperthyroidism or thyrotoxicosis other than GD, had undergone treatments for hyperthyroidism other than methimazole, had received subsequent radioiodine therapy or were taking statins or other medications that could affect lipid profiles.

Descriptive analysis was performed by presenting frequency tables for categorical variables and measures of central tendency and dispersion for numerical variables. When appropriate, proportions were compared using the chi-square test. The Kruskal–Wallis test was applied for comparing numerical measurements across three groups, followed by Dunn’s test to identify specific differences when necessary. To compare numerical measurements between two groups, the Mann–Whitney U test was applied. Simple ordinal logistic regression and multiple regression were employed to identify factors associated with disease severity according to the two criteria. The variable selection process followed a stepwise method. A significance level of 5% was adopted for all statistical tests. All analyses were conducted using SAS for Windows, version 9.4 (SAS Institute Inc., USA; https://www.sas.com).

## Results

The initial cohort of patients diagnosed with GD comprised 339 individuals. Of these, 211 patients were treated with methimazole and included in the study. Following the application of inclusion and exclusion criteria, the final cohort was refined to 165 patients. Table 1 presents an analysis of the general characteristics observed within this group (clinical and laboratory aspects in this population).

**Table 1:** General characteristics of the patients analyzed with GD. Data are presented as mean ± SD or as *n* (%).

Variables	Values
Clinical features	
Male	26 (15.8%)
Female	139 (84.2%)
Age	46.51 ± 14.34
BMI	28.61 ± 5.70
Smoking status	
Current smoker	24 (14.9%)
Nonsmoker	103 (64.0%)
Former smoker	34 (21.1%)
Thyroid evaluation	
TSH, mIU/L	1.92 ± 1.22
FT4, ng/dL	1.24 ± 0.68
Thyroid US volume, mL	18.47 ± 10.12
TRAb, IU/L	7.28 ± 10.35
Positive	99 (62.7%)
TPOAb	
Positive	108 (69.7%)
TgAb	
Positive	65 (42.2%)
Cholesterol evaluation	
TC (mg/dL)	192.12 ± 39.56
HDL cholesterol (mg/dL)	54.23 ± 14.30
LDL cholesterol (mg/dL)	118.27 ± 34.21
Triglycerides (mg/dL); *n* = 62	106.93 ± 50.78
Group evaluation	
CAS (categorized)	
Group A – CAS 0	80 (48.5%)
Group B – CAS 1 and CAS 2	56 (33.9%)
Group C – CAS 3 or more	29 (17.6%)
EUGOGO (categorized)	
Group A – absent disease	81 (49.1%)
Active	0
Nonactive	81 (100%)
Group B – mild	52 (31.5%)
Active	4 (7.7%)
Nonactive	48 (92.3%)
Group C – moderate-to-severe/sight-threatening	32 (19.4%)
Active	26 (81.2%)
Nonactive	6 (18.8%)

BMI, body mass index; TRAb, thyrotropin receptor antibodies; TPOAb, thyroid peroxidase antibodies; TgAb, thyroglobulin antibodies; TSH, thyroid-stimulating hormone; FT4, free thyroxine; US, ultrasound; HDL, high-density lipoprotein; LDL, low-density lipoprotein; CAS, clinical activity score; GD, Graves’ disease; TC, total cholesterol.

Comparative analyses were conducted across the groups using categorical data. When comparing groups based on the CAS, group A consisted of patients with a CAS of 0, group B included patients with a CAS of 1 or 2 and group C comprised patients with active disease, defined as a CAS of 3 or more (Table 2). To evaluate the severity of TED according to the EUGOGO classification, patients were divided into group A (no disease/absent), group B (mild forms) and group C (moderate-to-severe forms, including sight-threatening TED), as shown in Table 3.

**Table 2:** Descriptive analysis and comparisons between CAS groups. Data are presented as mean ± SD or as *n* (%).

Variable	Group A: CAS 0	Group B: CAS 1 + CAS 2	Group C: CAS ≥ 3	*P* value
General characteristics				
Male	11 (13.8%)	8 (14.3%)	7 (24.1%)	
Female	69 (86.3%)	48 (85.7%)	22 (75.9%)	
Age	44.35 ± 15.16 (*n* = 80)	46.38 ± 11.86 (*n* = 55)	52.72 ± 14.96 (*n* = 29)	0.0166*#
BMI	27.10 ± 5.73	29.72 ± 5.69	30.29 ± 4.04	ns
Smoking status				
Current smoker	7 (8.9%)	9 (16.7%)	8 (28.6%)	0.0225^†^
Nonsmoker	58 (73.4%)	34 (63.0%)	11 (39.3%)	
Former smoker	14 (17.7%)	11 (20.4%)	9 (32.1%)	
Thyroid evaluation				
TSH mIU/L	1.88 ± 1.10	2.02 ± 1.37	1.82 ± 1.27	ns
FT4 ng/dL	1.23 ± 0.34	1.16 ± 0.22	1.40 ± 1.49	ns
Thyroid US volume mL	18.20 ± 10.54	18.27 ± 10.07	20.14 ± 8.68	ns
TRAb IU/L	7.30 ± 10.49	7.24 ± 11.01	7.33 ± 8.58	ns
Positive	52 (65.8%) 30	30 (54.5%)	17 (70.8%)	ns
TPOAb				
Positive	57 (73.1%)	33 (62.3%)	18 (75.0%)	ns
TgAb				
Positive	36 (46.2%)	20 (37.7%)	9 (39.1%)	ns
Cholesterol evaluation				
Cholesterol (mg/dL)	185.42 ± 35.72	192.44 ± 42.89	210.43 ± 38.61	0.0095*#
HDL (mg/dL)	56.03 ± 13.27	52.57 ± 13.14	52.52 ± 18.53	ns
LDL (mg/dL)	112.33 ± 28.83	117.91 ± 37.78	135.17 ± 35.99	0.0055*
Triglycerides (mg/dL)	101.82 ± 52.91	107.65 ± 48.12	119.52 ± 49.02	ns

BMI, body mass index; TRAb, thyrotropin receptor antibodies; TPOAb, thyroid peroxidase antibodies; TgAb, thyroglobulin antibodies; TSH, thyroid-stimulating hormone; FT4, free thyroxine; US, ultrasound; HDL, high-density lipoprotein; LDL, low-density lipoprotein; CAS, clinical activity score; ns, not statistically significant.

# Differences between (Dunn test): 1 and 3; 2 and 3.

* Based on the Kruskal–Wallis test; #based on Dunn’s test.

† Based on chi-square test.

**Table 3:** Descriptive analysis and comparisons between the EUGOGO groups (group A: absent; group B: mild; group C: moderate-to-severe/sight-threatening). Data are presented as mean ± SD or as *n* (%).

Variable	Group A	Group B	Group C	*P* value
General characteristics				
Male	11 (13.6%)	7 (13.5%)	8 (25.0%)	ns
Female	70 (86.4%)	45 (86.5%)	24 (75.0%)	ns
Age	44.30 ± 15.08 (*n* = 81)	47.04 ± 12.88 (*n* = 52)	51.28 ± 13.79 (*n* = 32)	0.0460*#
BMI	27.04 ± 5.65	30.00 ± 5.80	29.63 ± 4.52	ns
Smoking status				
Current	7 (8.8%)	8 (16.3%)	9 (28.1%)	0.0443^†^#
Nonsmoker	58 (72.5%)	31 (63.3%)	14 (43.8%)	
Former smoker	15 (18.8%)	10 (20.4%)	9 (28.1%)	
Thyroid evaluation				
TSH mIU/L	1.86 ± 1.10	1.95 ± 1.34	1.99 ± 1.34	ns
FT4 ng/dL	1.24 ± 0.34	1.14 ± 0.21	1.40 ± 1.42	ns
Thyroid US volume mL	18.22 ± 10.47	18.46 ± 10.97	19.39 ± 6.58	ns
TRAb IU/L	7.26 ± 10.43	7.94 ± 11.32	6.10 ± 8.24	ns
Positive	53 (66.3%)	29 (56.9%)	17 (63.0%)	ns
TPOAb				
Positive	58 (73.4%)	32 (65.3%)	18 (66.7%)	ns
TgAb				
Positive	36 (45.6%)	19 (38.8%)	10 (38.5%)	ns
Cholesterol evaluation				
Cholesterol (mg/dL )	185.23 ± 35.53	190.27 ± 42.19	212.97 ± 39.09	0.0025*^#^
HDL (mg/dL)	56.09 ± 13.20	53.40 ± 13.61	50.91 ± 17.46	0.0436*^#^
LDL (mg/dL)	112.08 ± 28.74	114.90 ± 35.72	139.25 ± 37.10	0.0006*^#^
Triglycerides (mg/dL)	101.88 ± 52.58	107.14 ± 52.82	119.25 ± 41.37	0.0393*^#^

BMI, body mass index; TRAb, thyrotropin receptor antibodies; TPOAb, thyroid peroxidase antibodies; TgAb, thyroglobulin antibodies; TSH, thyroid-stimulating hormone; FT4, free thyroxine; US, ultrasound; HDL, high-density lipoprotein; LDL, low-density lipoprotein; ns, not statistically significant.

# Differences between (Dunn test): 1 and 3; 2 and 3.

* Based on the Kruskal–Wallis test; #based on Dunn’s test.

† Based on chi-square test.

In euthyroid patients, several variables were analyzed, including gender, body mass index (BMI), smoking status, thyroid antibodies, TSH, fT4, cholesterol levels and triglycerides. The analysis of the study population revealed a significant association between low-density lipoprotein (LDL) cholesterol and TC levels with TED activity, as measured by the CAS (*P* = 0.007, OR: 1.012, 95% CI: 1.003–1.021 and *P* = 0.010, OR: 1.010, 95% CI: 1.002–1.018, respectively) and TED severity according to EUGOGO standards (*P* = 0.001, OR: 1.015, 95% CI: 1.006–1.024 and *P* = 0.004, OR: 1.011, 95% CI: 1.004–1.019). The logistic regression model corroborated these findings, as detailed in Tables 4 and 5, when the three groups were compared.

**Table 4:** Results of simple and multiple logistic regressions, proportional odds model, for studying the CAS (modeling the group probability of CAS groups – group A: CAS = 0, *n* = 78; group B: CAS = 1 or 2, *n* = 52; or group C: CAS ≥ 3, *n*=27).

Variable/category	*P*-value	OR	95% CI
Simple analysis			
Age	0.0098	1.028	1.007–1.050
Thyroid US volume mL	ns	ns	ns
BMI	0.0484	1.084	1.001–1.174
TRAb	ns	ns	ns
Negative vs positive	ns	ns	ns
FT4	ns	ns	ns
TSH	ns	ns	ns
Cholesterol	0.0102	1.010	1.002–1.018
HDL	ns	ns	ns
LDL	0.0071	1.012	1.003–1.021
Triglycerides	ns	ns	ns
TPOAb			
Negative vs positive	ns	ns	ns
TgAb			
Negative vs positive	ns	ns	ns
Smoking status			
Smoker vs nonsmoker	0.0043	3.408	1.469–7.908
Former vs nonsmoker	ns	ns	ns
Multiple analysis*			
LDL	0.0112	1.012	1.003–1.021
Smoking status			
Smoker vs nonsmoker	0.0110	3.096	1.296–7.394
Nonsmoker vs former	0.1011	1.873	0.885–3.966

BMI, body mass index; TRAb, thyrotropin receptor antibodies; TPOAb, thyroid peroxidase antibodies; TgAb, thyroglobulin antibodies; TSH, thyroid-stimulating hormone; FT4, free thyroxine; US, ultrasound; HDL, high-density lipoprotein; LDL, low-density lipoprotein; CAS, clinical activity score; OR, odds ratio; ns, not statistically significant; 95% CI, 95% confidence interval.

* Stepwise selection process.

**Table 5:** Results of simple and multiple logistic regressions, proportional odds model, for the EUGOGO study (modeling the group probability of EUGOGO groups – group A: absent, *n* = 79; group B: mild, *n* = 45; or group C: moderate-to-severe/sight-threatening, *n* = 30).

Variable/category	*P*-value	OR	95% CI
Simple analysis			
Age	0.0187	1.026	1.004–1.047
Sex			
Male vs female	ns	ns	ns
Thyroid US volume mL	ns	ns	ns
BMI	ns	ns	ns
TRAb	ns	ns	ns
Negative vs positive	ns	ns	ns
FT4	ns	ns	ns
TSH	ns	ns	ns
Cholesterol	0.0042	1.011	1.004–1.019
HDL	0.0612	0.979	0.958–1.001
LDL	0.0013	1.015	1.006–1.024
Triglycerides	ns	ns	ns
TPOAb			
Negative vs positive	ns	ns	ns
TgAb			
Negative vs positive	ns	ns	ns
Smoking status			
Smoker vs nonsmoker	0.0047	3.347	1.448–7.738
Former vs nonsmoker	ns	ns	ns
Multiple analysis*			
HDL	0.0384	0.973	0.948–0.999
LDL	0.0054	1.013	1.004–1.023
Smoking status			
Smoker vs nonsmoker	0.0189	2.881	1.190–6.971
Nonsmoker vs former	ns	ns	ns

BMI, body mass index; TRAb, thyrotropin receptor antibodies; TPOAb, thyroid peroxidase antibodies; TgAb, thyroglobulin antibodies; TSH, thyroid-stimulating hormone; FT4, free thyroxine; US, ultrasound; HDL, high-density lipoprotein; LDL, low-density lipoprotein; CAS, clinical activity score; OR, odds ratio; 95% CI, 95% confidence interval.

* Stepwise selection process.

Results from the multiple regression analyses indicated that greater TED activity was significantly associated with higher LDL cholesterol levels (*P* = 0.011, OR: 1.012, 95% CI: 1.003–1.021) and smoking status (*P* = 0.011, OR: 3.096, 95% CI: 1.296–7394), as demonstrated in Table 4. Furthermore, TED severity was significantly correlated with lower HDL cholesterol levels (*P* = 0.038, OR: 0.973, 95% CI: 0.948–0.999), elevated LDL cholesterol levels (*P* = 0.054, OR: 1.013, 95% CI: 1.004–1.023) and active smoking status (*P* = 0.019, OR: 2.881, 95% CI: 1.190–6.971), as shown in Table 5.

Continuing with the comparative analysis among the three groups, a simple logistic regression analysis indicated an association between BMI and CAS, suggesting that a higher BMI may be linked to an increased CAS (BMI: *P* = 0.0484; OR: 1.084). However, this association was not significant in further analysis. Age, in contrast, showed no statistically significant correlation with CAS in either the simple or multivariate analyses. When EUGOGO criteria were applied, age was significant in simple logistic regression analysis (age: *P* = 0.0187, OR: 1.026); however, it was not significant in multiple logistic regression analysis. No association with BMI was identified when evaluating TED according to the EUGOGO criteria.

On the other hand, when analyses were performed comparing the groups with inactive disease (group B – CAS 1 and CAS 2) and active disease (group C – CAS ≥ 3), excluding the control group (patients without TED) from the analysis, a significant association between LDL cholesterol and age with TED activity was observed. This was confirmed through simple logistic regression for age (*P* = 0.04469, OR: 1.040, 95% CI: 1.001–1.081), as shown in Table 6. However, when evaluated using multiple regression, no variable reached statistical significance at the 5% level.

**Table 6:** Descriptive analysis and comparisons between CAS group B (CAS 1 and 2) and group C (CAS ≥ 3). Data are presented as mean ± SD or as *n* (%).

Variable	Group B	Group C	*P* value
General characteristics			
Male/female	8 (14.5%)	7 (24.1%)	
Female	47 (85.5%)	22 (75.9%)	
Age	46.50 ± 11.93 (*n* = 54)	52.72 ± 14.96 (*n* = 29)	0.0207*
BMI	29.85 ± 5.72	30.29 ± 4.04	ns
Smoking status			
Current	9 (16.7%)	8 (28.6%)	ns
Nonsmoker	34 (63.0%)	11 (39.3%)	
Former smoker	11 (20.4%)	9 (32.1%)	
Thyroid evaluation			
TSH mIU/L	2.04 ± 1.37	1.82 ± 1.27	ns
FT4 ng/dL	1.16 ± 0.22	1.40 ± 1.49	ns
Thyroid US volume mL	18.23 ± 10.17	20.14 ± 8.68	ns
TRAb IU/L	7.24 ± 11.01	7.33 ± 8.58	ns
Positive	29 (53.7%)	17 (70.8%)	ns
TPOAb			
Positive	32 (61.5%)	18 (75.0%)	ns
TgAb			
Positive	20 (38.5%)	9 (39.1%)	ns
Cholesterol evaluation			
Cholesterol (mg/dL)	192.85 ± 43.18	210.43 ± 38.61	0.0528*
HDL (mg/dL)	52.42 ± 13.21	52.52 ± 18.53	ns
LDL (mg/dL)	118.38 ± 37.97	135.17 ± 35.99	0.0273*
Triglycerides (mg/dL)	107.68 ± 48.58	119.52 ± 49.02	ns

BMI, body mass index; TRAb, thyrotropin receptor antibodies; TPOAb, thyroid peroxidase antibodies; TgAb, thyroglobulin antibodies); TSH, thyroid-stimulating hormone; FT4, free thyroxine; US, ultrasound; HDL, high-density lipoprotein; LDL, low-density lipoprotein; CAS, clinical activity score.

* Based on Mann–Whitney test.

In addition, a specific analysis of TED patients classified by the EUGOGO criteria was conducted. Groups B (mild forms of TED) and C (moderate-to-severe forms, including sight-threatening TED) were compared. The analysis revealed a significant association between disease severity and higher levels of LDL cholesterol (*P* = 0.0062, OR: 1.014, 95% CI: 1.005–1.033) and TC (*P* = 0.0215, OR: 1.014, 95% CI: 1.002–1.026). These findings were further supported by the multiple logistic regression model for LDL cholesterol (*P* = 0.0145, OR: 1.017, 95% CI: 1.003–1.031). The data from these analyses, based on the EUGOGO classification, are presented in Table 7.

**Table 7:** Descriptive analysis and comparisons between EUGOGO group B (mild) and group C (moderate-to-severe/sight-threatening). Data are presented as mean ± SD or as *n* (%).

Variable	Group B	Group C	*P* Value
General characteristics			
Male	7 (13.5%)	8 (25.0%)	ns
Female	45 (86.5%)	24 (75.0%)	ns
Age	47.04 ± 12.88 (*n* = 52)	51.28 ± 13.79 (*n* = 32)	ns
BMI	30.00 ± 5.80	29.63 ± 4.52	ns
Smoking status			
Current	8 (16.3%)	9 (28.1%)	ns
Nonsmoker	31 (63.3%)	14 (43.8%)	
Former smoker	10 (20.4%)	9 (28.1%)	
Thyroid evaluation			
TSH mIU/L	1.95 ± 1.34	1.99 ± 1.34	ns
FT4 ng/dL	1.14 ± 0.21	1.40 ± 1.42	ns
Thyroid US volume mL	18.46 ± 10.97	19.39 ± 6.58	ns
TRAb IU/L	7.94 ± 11.32	6.10 ± 8.24	ns
Positive	29 (56.9%)	17 (63.0%)	ns
TPOAb			
Positive	32 (65.3%)	18 (66.7%)	ns
TgAb			
Positive	19 (38.8%)	10 (38.5%)	ns
Cholesterol evaluation			
Cholesterol (mg/dL)	190.27 ± 42.19	212.97 ± 39.09	0.0132*
HDL (mg/dL)	53.40 ± 13.61	50.91 ± 17.46	ns
LDL (mg/dL)	114.90 ± 35.72	139.25 ± 37.10	0.0024*
Triglycerides (mg/dL)	107.14 ± 52.82	119.25 ± 41.37	ns

BMI, body mass index; TRAb, thyrotropin receptor antibodies; TPOAb, thyroid peroxidase antibodies; TgAb, thyroglobulin antibodies; TSH, thyroid-stimulating hormone; FT4, free thyroxine; US, ultrasound; HDL, high-density lipoprotein; LDL, low-density lipoprotein.

* Based on Mann–Whitney test.

## Discussion

Multiple molecular mechanisms can contribute to the development of TED. These include humoral and cell-mediated immune responses, cytokine production (such as TNF-alpha, IL-1 beta, IL-6 and CCL20) and oxidative stress (1, 2, 16). These factors impact the fibroadipose tissue within the orbit, leading to structural changes.

The condition likely arises from an immune response against autoantigens present in fibroblasts and thyroid tissue cells in the orbit, with the thyroid-stimulating hormone receptor serving as a significant autoantigen in both instances. In addition, research has explored the role of the insulin-like growth factor-1 receptor (IGF-1R) and its connection with the progression of immune events (1).

This discussion has emphasized the roles of inflammation and oxidative stress. The involvement of oxidative stress in the disease’s pathophysiology has been debated across various contexts. Fischli *et al.* (17) suggested that smoking can influence levels of ferritin, hepcidin and thyroid hormones in patients with GD, thereby contributing to TED’s development. *In vitro* studies have shown that cigarette smoke increases free radical production while reducing antioxidants. These findings indicate that smoking is associated with changes in orbital tissue, either through direct exposure or via systemic circulation (4, 17, 18, 19).

Stasiak *et al.* (20) identified patterns linking high-risk HLA (human leukocyte antigen) alleles for TED with TC levels. The study found significantly increased TC levels in carriers of two high-risk TED alleles, specifically HLA-B37:01 and HLA-C03:02. For the latter allele, LDL concentrations were also significantly higher in carriers compared to noncarriers. Although further research is needed, these findings underscore the importance of TC and LDL levels in assessing TED risk, suggesting that the associations between cholesterol levels and TED may be HLA dependent (20).

The relationship between TED and cholesterol might involve an inflammatory response triggered by elevated cholesterol levels (21, 22). Disorders in lipid profiles often correlate with mild-to-moderate chronic systemic inflammation (23). Excess free fatty acid load in liver cells, disrupted mitochondrial function and alterations in the endoplasmic reticulum can lead to the production of reactive oxygen species.

Moreover, increased free fatty acid metabolism can trigger the release of proinflammatory cytokines, particularly interleukin-6 and TNF-alpha, which play key roles in TED development (18, 24). Cholesterol may contribute to tissue inflammation by promoting the assembly of Toll-like receptor 4-myeloid differentiation factor 2 (TLR4-MD2) and TLR4-CD14 complexes, enhancing the response to TLR4 ligands such as lipopolysaccharides (21, 22).

A recent study analyzing the full genome of orbital fibroblasts from TED patients noted a trend toward TLR4 gene overexpression (1.58-fold increase) in these cells compared to controls, although the results did not reach statistical significance, possibly due to the limited sample size. In addition, there was a decrease in LDL receptor gene expression (1.64-fold decrease), reinforcing the link between elevated cholesterol and TED. These findings align with the well-documented association between high cholesterol levels and TED (25, 26).

The hypothesis that statin treatment may serve as a protective factor has garnered significant attention recently. Studies on cardiovascular protection have demonstrated the pleiotropic antiinflammatory effects of statins, independent of their cholesterol-lowering properties, suggesting that statins could be employed as novel adjunctive antiinflammatory therapy. Research on signaling proteins, such as those in the CCN1 family, supports this theory, indicating that specific drugs might inhibit the inflammatory cascade in certain contexts (27, 28, 29).

Koch *et al.* proposed that statins could shift a predominantly proinflammatory T cell response to an antiinflammatory one by increasing the expression of TH2 cells and regulatory T cells. Similarly, they suggested that statins might help redirect disease-specific proinflammatory T cells from the affected area to the bloodstream, potentially leading to clinical remission (30).

Stein *et al.* studied patients recently diagnosed with GD and found that the risk of developing TED (16) decreased when patients were treated with thyroidectomy or statin use. Their multivariate regression model revealed that the combination of antithyroid drugs with radiotherapy did not reduce TED risk as effectively as thyroidectomy. The study also discussed the impact of statin use; patients introduced to COX-2 inhibitors and statins who used these drugs for at least 60 days experienced an approximately 40% reduction in TED risk.

Lanzolla *et al.* and Sabini *et al.* demonstrated a correlation between TED and increased levels of TC and LDL in an Italian population. The relationship between LDL levels and TED symptoms appears more clearly defined than that between TC and TED. Comparative studies considering factors such as age, ethnicity and the duration of hyperthyroidism have shown significantly higher LDL cholesterol and TC prevalence in TED patients compared to those without TED (21, 22, 23).

Between June 1, 2020, and November 30, 2020, Lanzolla and colleagues conducted a randomized, open-label, phase 2, adaptive clinical trial at a single tertiary referral hospital in Italy. Patients with moderate-to-severe, active Graves’ orbitopathy were treated with intravenous glucocorticoids (ivGC) versus a group treated with ivGC plus oral atorvastatin. They observed an improvement in TED outcomes in patients with moderate-to-severe, active TED who were hypercholesterolemic (31, 32).

Various population studies, including those conducted in Japan, North America and Denmark (2), have suggested a correlation between advanced age and the severity of orbitopathy, a conclusion also supported by our research. A North American study revealed a notable pattern with two peaks of TED prevalence – one at 40–44 years and another at 60–64 years for women and 45–49 years and 65–69 years for men (2). This observation was further confirmed by a Japanese study involving 10,931 patients, which found the average onset age of TED to be 39 years in women and 43 years in men.

In addition, an Italian study found no significant difference in average age between GD patients without TED and those with mild TED (46 and 44 years, respectively). However, there was a significant increase in the average age of patients with moderate-to-severe TED (54 years). The age-dependent severity of TED was further emphasized in a Danish study, which observed that the average age of patients with moderate-to-severe TED was 50 years before salt iodization and increased to 56 years afterward. Moreover, the study found that patients under 40 years of age had a reduced risk of developing moderate-to-severe TED (2).

Despite Brazil’s ethnic diversity, our study aligns with the existing literature, revealing a comparable positive association between elevated LDL and TC levels and TED. This correlation links increased lipid levels to disease activity and severity. Furthermore, our findings underscore the associations between advancing age, smoking habits and the progression of TED. However, due to the study’s retrospective design, it is subject to certain biases, including those related to the timing of TC and LDL measurements.

In conclusion, our study reinforces the link between high LDL levels and TED activity and severity in the Brazilian population, contributing to a better understanding of potential risk factors associated with the disease. The role of oxidative stress and inflammation in TED onset underscores the need for new treatment approaches, including the potential use of statins as adjunctive antiinflammatory therapy. Nevertheless, further research is required to validate these findings and explore the potential benefits of statin use in managing TED.

## Declaration of interest

The authors declare that there is no conflict of interest that could be perceived as prejudicing the impartiality of the research reported.

## Funding

This research did not receive any specific grant from any funding agency in the public, commercial or not-for-profit sector.

## Ethics statement

The study was submitted to the ethics committee and approved in December 2020 (CAAE number 39062520800005481). Written consent has been obtained from each patient after full explanation of the purpose and nature of all procedures used.
